# The importance of age in compositional and functional profiling of the human intestinal microbiome

**DOI:** 10.1371/journal.pone.0258505

**Published:** 2021-10-18

**Authors:** Elio L. Herzog, Melania Wäfler, Irene Keller, Sebastian Wolf, Martin S. Zinkernagel, Denise C. Zysset-Burri

**Affiliations:** 1 Department of Ophthalmology, Inselspital, Bern University Hospital, University of Bern, Bern, Switzerland; 2 Graduate School for Cellular and Biomedical Sciences, University of Bern, Bern, Switzerland; 3 Department for BioMedical Research, University of Bern, Bern, Switzerland; 4 Interfaculty Bioinformatics Unit and Swiss Institute of Bioinformatics, University of Bern, Bern, Switzerland; University of Illinois Urbana-Champaign, UNITED STATES

## Abstract

The intestinal microbiome plays a central role in human health and disease. While its composition is relatively stable throughout adulthood, the microbial balance starts to decrease in later life stages. Thus, in order to maintain a good quality of life, including the prevention of age-associated diseases in the elderly, it is important to understand the dynamics of the intestinal microbiome. In this study, stool samples of 278 participants were sequenced by whole metagenome shotgun sequencing and their taxonomic and functional profiles characterized. The two age groups, *below65* and *above65*, could be separated based on taxonomic and associated functional features using Multivariate Association of Linear Models. In a second approach, through machine learning, biomarkers connecting the intestinal microbiome with age were identified. These results reflect the importance to select age-matched study groups for unbiased metagenomic data analysis and the possibility to generate robust data by applying independent algorithms for data analysis. Furthermore, since the intestinal microbiome can be modulated by antibiotics and probiotics, the data of this study may have implications on preventive strategies of age-associated degradation processes and diseases by microbiome-altering interventions.

## Introduction

Genetic diversity between humans does not only arise from allele frequency differences of shared human genes, but also from the vast number of genetic and metabolic diversity in intestinal microbial communities. The human intestinal microbiome is a complex system consisting of trillions of microorganisms that contribute to numerous functions of the host. Fermentation of indigestible food components, stimulation and regulation of the immune system, strengthening of the intestinal barrier and protection against pathogens [1] are some of the key functions of the intestinal microbiome. Despite its crucial role in human health, the composition of the intestinal microbiome is not uniform between individuals and populations [2]. Although there are significant differences in relative abundances of bacteria between individuals, the phyla *Bacteroidetes*, *Firmicutes* and *Proteobacteria* seem to dominate the composition in almost all individuals [3]. However, despite being able to remain stable over decades in an individual [4], the composition of the human intestinal microbiome is influenced by several factors, including the genetic background, gut architecture, immune system, body mass index (BMI), diet, life style, antibiotics intake, disease and age [5].

With increasing age, physiological functions of human organs start to gradually decrease [6], making them prone to infections and diseases and leading to a higher mortality risk in the elderly population [7, 8]. The gastrointestinal tract is also vulnerable to this aging process. Thus, understanding its age-related dynamics may be crucial for disease prevention in the elderly. The influence of age on the microbial composition in the gut has been investigated in many studies for over a decade [9–13]. The most noticeable feature in the microbiota of elderly individuals is an altered ratio of *Firmicutes* to *Bacteroidetes*, with an increased proportion of *Bacteroidetes* in the elderly [10]. This ratio has been shown to be of significant relevance in several disease states [14] and seems to be not only an effect of the current age, but can, in turn, have an impact on the ageing process itself [15, 16].

In this study, we aimed to investigate age-associated changes in the intestinal microbial composition and in microbial functional profiles. Stool samples of 278 participants were sequenced and analyzed using two independent approaches to identify associations between microbial abundances as well as functional profiles and age. In contrast to many previous studies, whole metagenome shotgun sequencing instead of 16S ribosomal RNA sequencing was applied, allowing the identification of archaea, viruses and eukaryotes in addition to bacteria. Since the stability of the intestinal microbiome diminishes between an age of 63 and 76 years [12, 13], we set a threshold of 65 years to divide the cohort into two sex-matched age groups *below65* and *above65*.

## Materials and methods

### Study design and recruitment

Participants (n = 278) were recruited from the Department of Ophthalmology at the University Hospital Bern (Inselspital), Switzerland. The study follows the ethical principles for medical research found in the Declaration of Helsinki and was approved by the Ethics Committee of the Canton of Bern (Clinical-Trails.gov: NCT02438111). After receiving oral and written information, all participants signed the informed consent prior to participation. We tested for differences between study groups in a range of demographic values using either Welch’s t-test (for age and BMI) or Fisher’s exact test (for sex and smoking; Table 1). Exclusion criteria were chronic inflammatory and gastrointestinal diseases (including previous surgery in the gastrointestinal tract) as well as systemic antibiotic treatment within the last three months. All participants were Caucasian and were 18 years of age or older.

**Table 1:** Characterization of the study cohort.

Feature	above 65	below 65	p-value
N	145	133	
Sex (% male)	64 (44.1)	67 (50.4)	0.357
Age [years] (mean (SD), min, max	77.25 (6.32), 65.07, 92.94	49.69 (12.05), 19.55, 64.71	<0.001
BMI [kg/m^2^] (mean (SD))	26.10 (4.54)	24.80 (4.44)	0.016
Smoker (%)			0.058
No	85 (58.6)	74 (55.6)	
Previous	53 (36.6)	42 (31.6)	
Yes	7 (4.8)	17 (12.8)	

BMI = Body Mass Index [kg/m^2^]. Differences in BMI and age were calculated using Welch’s t-test, differences in sex and smoking through Fisher’s exact test.

### Sample collection, data sequencing and quality control

Chilled stool samples were collected and delivered in an aerobic environment and brought to the laboratory within 16 hours after defecation. Upon arrival, they were immediately frozen at -20°C. Following the manufacture’s protocol, the PSP®Spin Stool DNA Plus kit (Stratec Biomedical AG, Beringen, Switzerland) with an integrated RNA digestion step using 100 mg/ml RNase A (Qiagen, Homberchtikon, Switzerland) was used to isolate metagenomic DNA from up to 200 mg stool sample. Whole metagenome shotgun sequencing was performed at BGI Europe (Copenhagen N, Denmark) and the Next Generation Sequencing Platform of the University of Bern, Switzerland. For library preparation, the TruSeq DNA PCR-Free Library Preparation kit was used. Cluster generation and sequencing were done following standard pipelines of Illumina HiSeq 3000 platforms, resulting in 150bp paired-end reads. Quality filtering was performed using Trimmomatic v.0.32 to remove adapter sequences and reads shorter than 70bp and to trim low-quality bases from both ends [17]. Resulting reads were mapped against hg19 human reference genome to identify sequences of human origin using Bowtie2 (v.2.2.4) [18]. Reads of human origin were excluded, resulting in non-human, high-quality reads for further analysis.

### Microbial and functional profiling of the intestinal microbiomes

The Metagenomic Phylogenetic Analysis tool (MetaPhlAn2, v.2.0–2.6.0) [19] and the marker database (v.20) using default settings were used to perform metagenomic profiling by mapping non-human high-quality reads to a set of clade-specific markers. Alignment was performed using Bowtie2 (v.2.2.4) followed by normalization of the total number of reads in each clade divided by the nucleotide length of its marker, resulting in the relative abundance of each taxonomic unit.

To detect the metabolic potential of the gut microbiome, the HMP (Human Microbiome Project [20]) Unified Metabolic Analysis Network (HUMAnN2, v.0.2.1 – v.0.11.0) [21] was applied for each sample separately with default settings based on the taxonomic profiles from MetaPhlAn2. Mapping reads to ChocoPhlAn, a functionally annotated pan-genome database, was performed with the help of Bowtie2 (v.2.2.4). To identify unmapped reads, Diamond (v.0.8.37) [22] in combination with the universal protein reference database UniRef90 [23] was used. The assignment of the resulting organism-specific gene hits to pathways was done through maximum parsimony using MinPath (v.1.2) [24]. Using this information, HUMAnN2 returned a list of genes and pathways and their relative abundances.

A Principle Component Analysis (PCA) between groups was computed and visualized using the function *prcomp* (data, center = T, scale = T) [25] and the library factoextra in the R software (version 3.6.0) [26]. PCA was performed for microbial abundances (Fig 3A) and pathway abundances (Fig 3B). The p-value for separation was assessed by Permutation Multi-variate Analysis of Variance (PERMANOVA) with 10’000 iterations using the R package vegan51 [27]. To find age-related taxonomic and functional features, Multivariate Association of Linear Models (MaAsLin) in the R package Maaslin (v.0.0.5) was used with default settings [28]. Moreover, associations of biological variables including sex and BMI with microbial and functional abundances were analyzed with MaAsLin. Differences were considered to be significant if the p-value < 0.05 and q-value < 0.2. MetaPhlAn2 abundance files were normalized by summing all values across all taxonomic levels for each participant followed by dividing each value by this sum. Selection was performed by maintaining only the most specific taxa if abundances of one clade of several taxonomic levels were identical for all participants.

### Group separation by machine learning

A machine learning approach was used to investigate a potential separation of the study groups *below65* and *above65* by their microbial and functional profiles and to find the main contributors of such a separation. Model selection for the best performing machine learning algorithm of the dataset was done with the R libraries mlbench (v.2.1) [29] and caret (v.6.0–84) [30], testing four common classification algorithms: CART (Classification and Regression Tree), SVM (Supported Vector Machines), RF (Random Forest) and KNN (K nearest neighbor). The best fitting model, R package randomForest (v.4.6–14), was consequentially used [31].

Parameter tuning of random forest was performed for mtry and nTree using random and grid search as well as build in tools. The algorithm’s performance on the whole dataset using 10 fold cross validation was calculated using the package caret (v.6.0–84) [30]. Cross-validation was repeated 10 times. Random forests performance was evaluated by fitting it on the training set using the fitted model and the function *predict* of the random Forest package. Receiver Operating Characteristic (ROC) curves were calculated using the R package ROCR (v.1.0–7) [32]. Shrinkage Discriminant Analysis (SDA) based on Correlation Adjusted T-scores (CAT-scores) was performed using the R package sda (v.1.3.7) [33]. A shrinkage CAT score between the mean values of the groups was computed for each predictor variable. The ranking for each feature was determined by a summary score (the weighted sum of squared CAT scores across classes) using microbial and pathway abundances as input. Based on Gene Ontology (GO) terms, functional features with top CAT scores were clustered using REVIGO [34].

## Results

### Taxonomic and functional characterization of the intestinal microbiota

**Fig 1:**
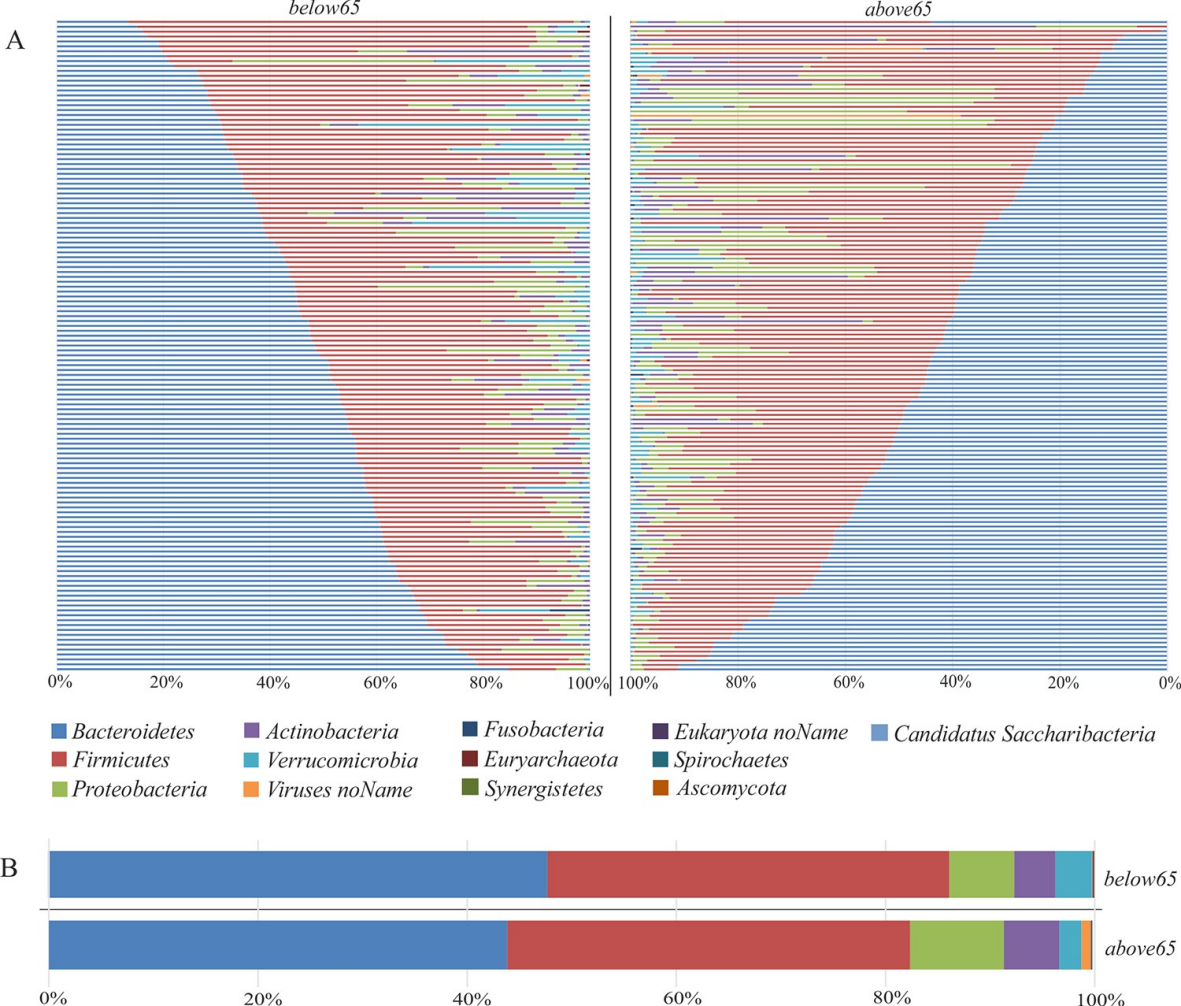
Taxonomic characterization of the intestinal microbiome. Relative abundances of microbiota at phylum level for each study subject (A) and averaged for study groups (B). *Above65* (patients aged 65 years and above; n = 145), *below65* (patients below 65 years of age; n = 133).

To find associations of age with functional and compositional alterations in the intestinal microbiome, the gut metagenomes of 278 study participants were sequenced. The cohort consisted of 145 participants aged equal or above 65 years (*above65*) and 133 participants aged below 65 years (*below65*) (Table 1). In total, 7.3 trillion 151 bp paired-end reads with an average of 26 ± 10.9 (SD) million reads per sample were generated. After trimming and filtering, 23 ± 9.9 (SD) million non-human high-quality reads per sample remained for further processing. Overall, 99.43% of the reads mapped to the bacterial kingdom (99.88% in participants below65, 99.01% in participants above65). *Bacteroidetes* and *Firmicutes*, followed by *Proteobacteria* and *Actinobacteria* were found to be the most abundant phyla (Fig 1B). Consistent with previous studies, *Bacteroidia* and *Clostridia* were the most abundant classes in the cohort [35]. The dominating genera were *Bacteroides*, *Alistipes* followed by *Subdoligranulum* and *Faecalibacterium*. An unclassified *Subdoligranulum* species, *Faecalibacterium prausnitzii*, *Alistipes putredinis*, *Prevotella copri* and *Bacteroides uniformis* were found to be the five most abundant species in the cohort (S1 Table). To describe the metabolic functions of these identified taxa, HUMAnN2 was applied on each sample separately, resulting in 793 assigned pathways.

### Classification of the microbiota into enterotypes

**Fig 2:**
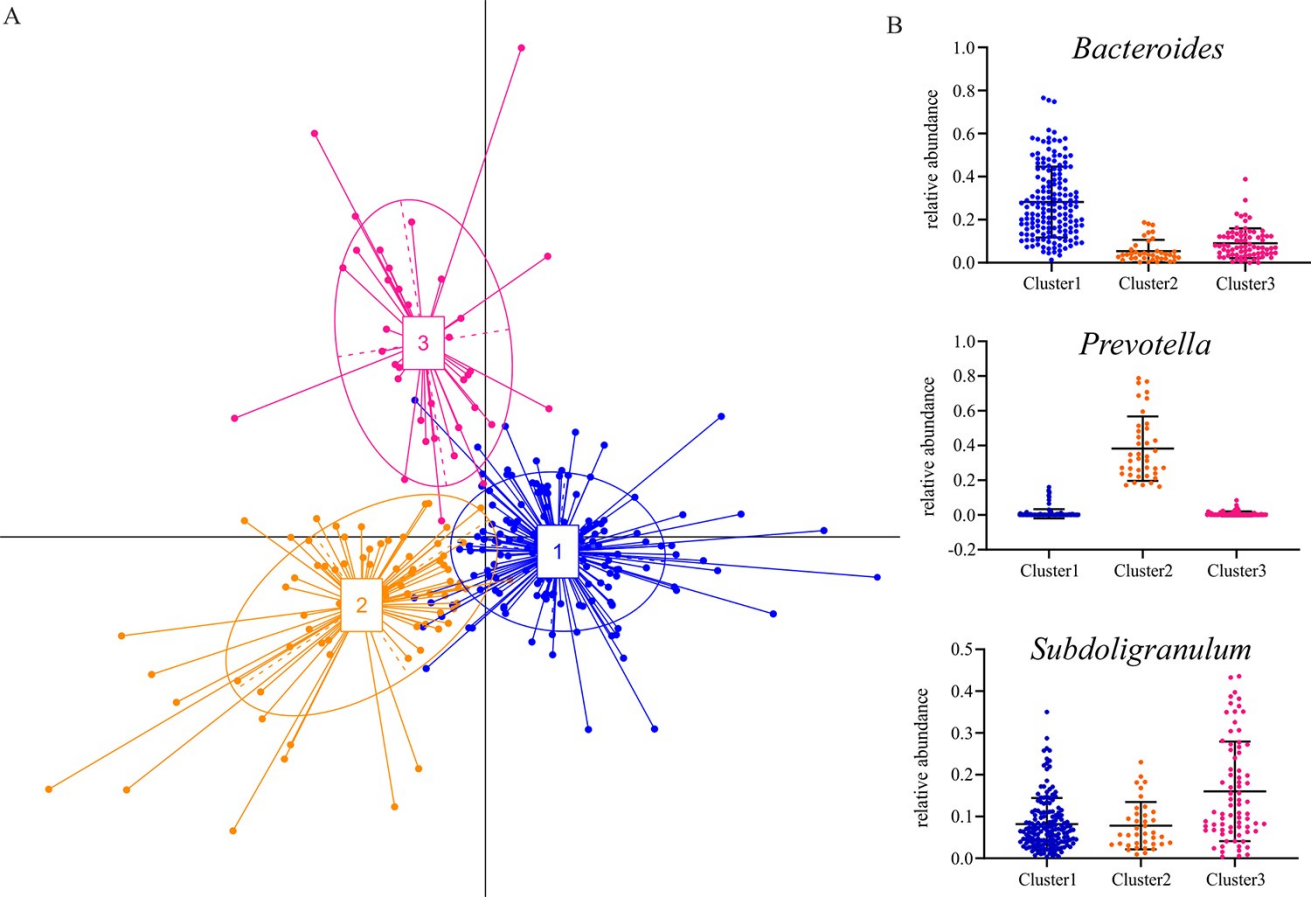
Intestinal microbial enterotypes. (A) Based on the abundance of microbial genera, three enterotypes were identified in the cohort using Between Class Analysis that visualizes results from Principal Component Analysis and clustering. (B) The relative abundances of the proposed drivers of these three enterotypes, the genera *Bacteroides*, *Prevotella* and *Subdoligranulum*, are shown for each subject.

In accordance with a previous study of Arumagam *et al*. [35], the intestinal microbiomes of our cohort could be divided into three enterotypes of distinct microbial composition. Out of the computed Jenson-Shannon distance of the genus abundances, clustering was done with Partitioning Around Medoids (PAM). The Calinski-Harabasz (CH) Index was used to determine that the optimal cluster number, i.e. returning the most robust partition of the dataset, was three clusters. Combining the results of a PCA and clustering through Between Class Analysis (BCA) resulted in graphical interpretation of the data in Fig 2A. In terms of abundance *Bacteroides*, *Prevotella* and *Subdoligranulum* were found to be the dominating genera in clusters 1, 2 and 3, respectively (Fig 2B). Applying Fisher’s exact test, subjects of group *above65* were over-represented in enterotype 2 (p = 0.0012) and under-represented in enterotype 3 (p = 0.0036), supposing an age-dependency of the proposed enterotypes.

### Age-dependent microbial and functional composition of the intestinal microbiota

**Fig 3:**
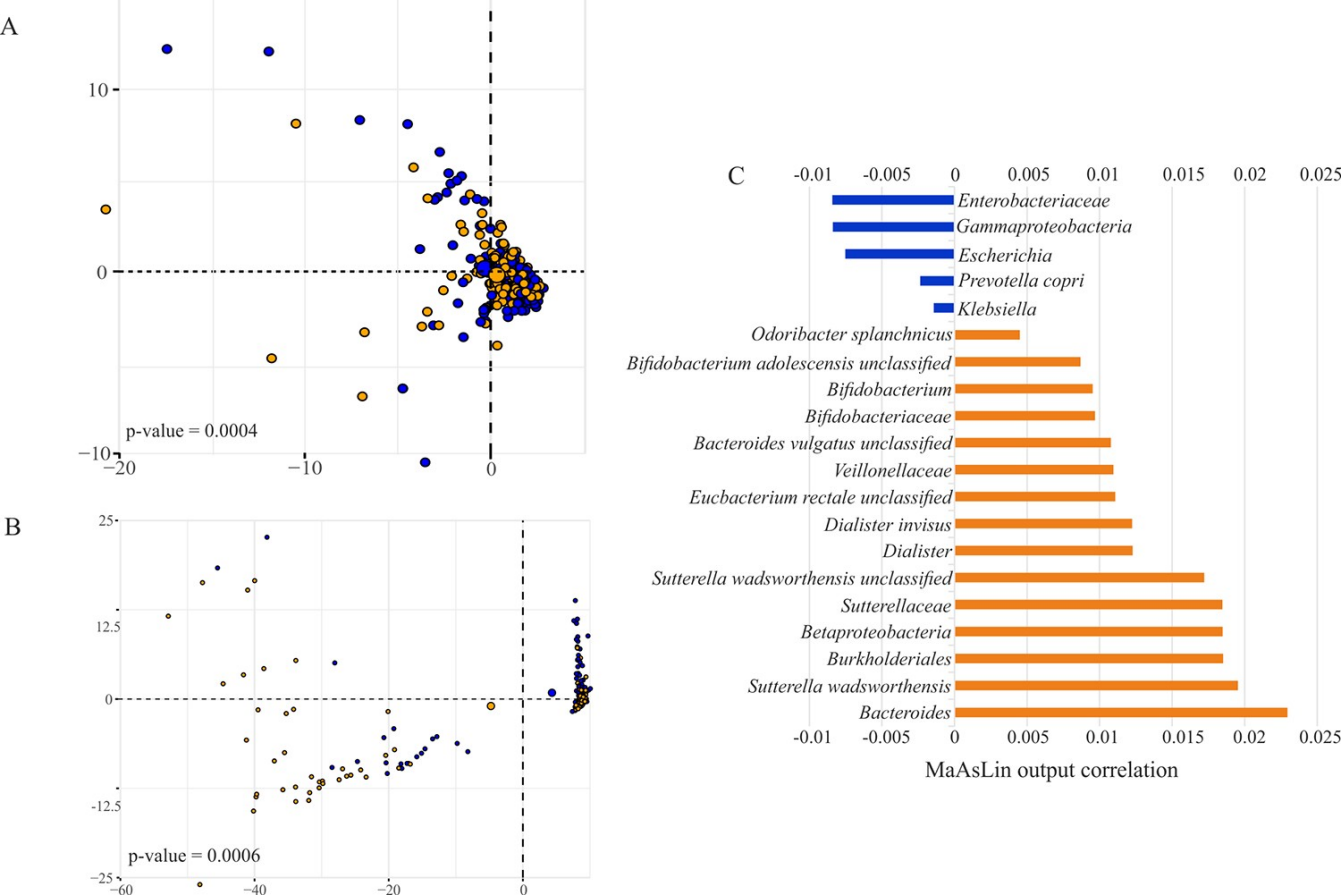
Distinct microbial and functional composition between age groups. Principal component analysis of (A) microbial and (B) pathway abundances separated the two age groups *above65* and *below65* (PERMANOVA, 10’000 iterations). Blue color represents *above65* (patients aged 65 years and above; n = 145), orange *below65* (patients below 65 years of age; n = 133). (C) Correlation between taxonomic features and age (MaAsLin, q < 0.2). Positive correlations (orange) imply higher abundance in below65, whereas negative correlations (blue) imply higher abundance in *above65*.

A PCA with age as grouping variable showed that differences in microbial species abundance as well as in pathway abundance separated the two age groups *above65* and *below65* with PERMANOVA confirming a significant p-value of 0.0004 and 0.0006, respectively (10’000 iterations; Fig 3A and 3B). To identify features that are different in relative abundance between the groups, MaAsLin was applied on the taxonomically and functionally profiled metagenomes. Out of the 20 identified taxa with age-dependence, 15 had a higher relative abundance in the *below65* group and five in the *above65* group (Fig 3C). Moreover, while the intestinal microbiomes of subjects of the *below65* group were enriched in genes of 248 pathways, microbiomes of subjects of the *above65* group were enriched in genes of 57 pathways (S2 Table). A boosting step in the MaAsLin algorithm ensures that only metadata that are associated with the given metagenomic feature are included in the model, implying that all associations detected by the modeling approach have been corrected for all other confounding factors. However, the effect of other biological variables including sex and BMI on the microbiome of this cohort was also investigated, showing that only a subspecies of *Bacteroidales bacterium ph8* was associated with sex with higher abundances in females compared to males and that the BMI positively correlated with the order *Selenomonadales* and negatively correlated with the family *Ruminococcaceae*. There was no association found between both, sex and BMI, and the functional profiles of the metagenomes in the cohort.

### Allocation of subjects to age groups based on the intestinal microbiome

**Fig 4:**
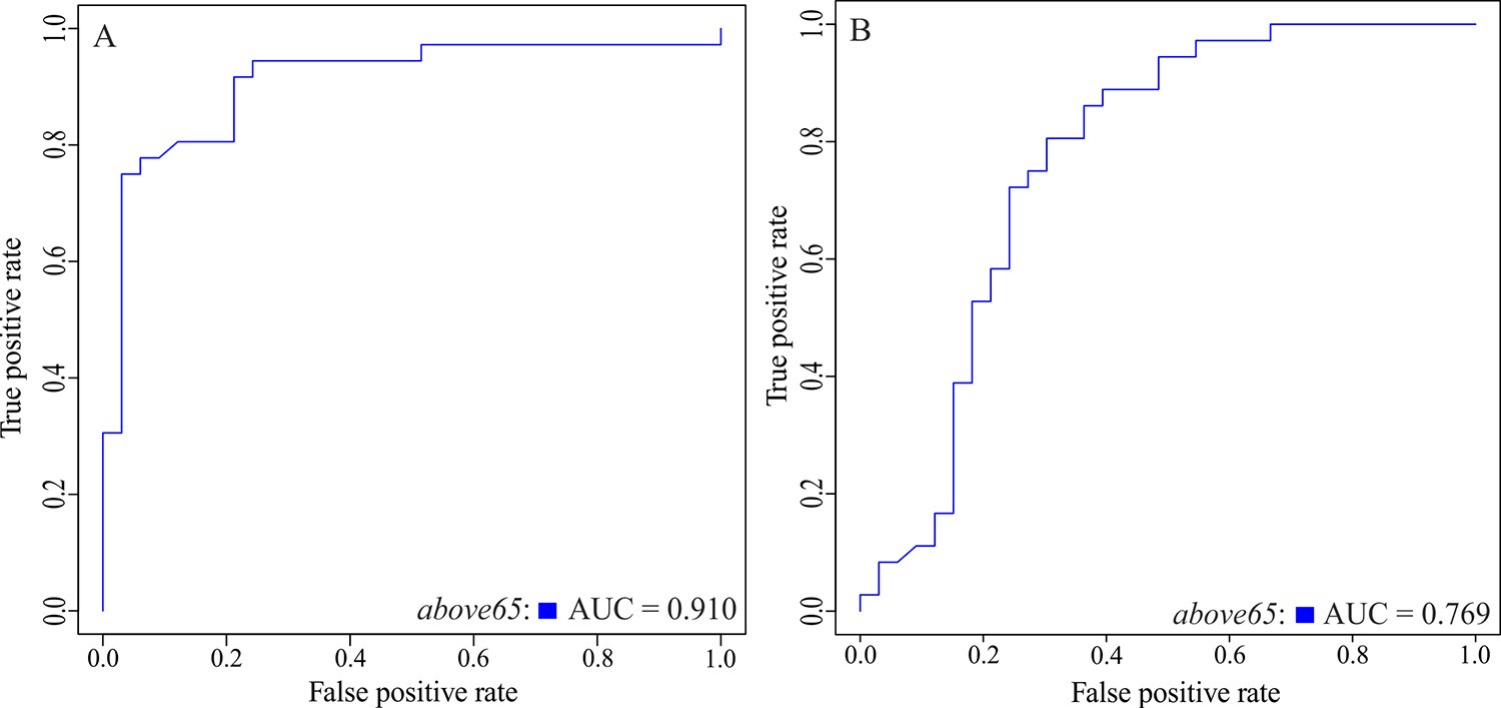
ROC curves for the random forest classifier for microbial abundances and pathway abundances. ROC curves visualizing the Random Forest classifier for (A) microbial abundances and (B) pathway abundances. The group *above65* is represented in blue (patients aged 65 years and above; n = 145), the curve for *below65* was omitted as its information is redundant. AUC, area under the curve; ROC, receiver operating characteristic.

**Fig 5:**
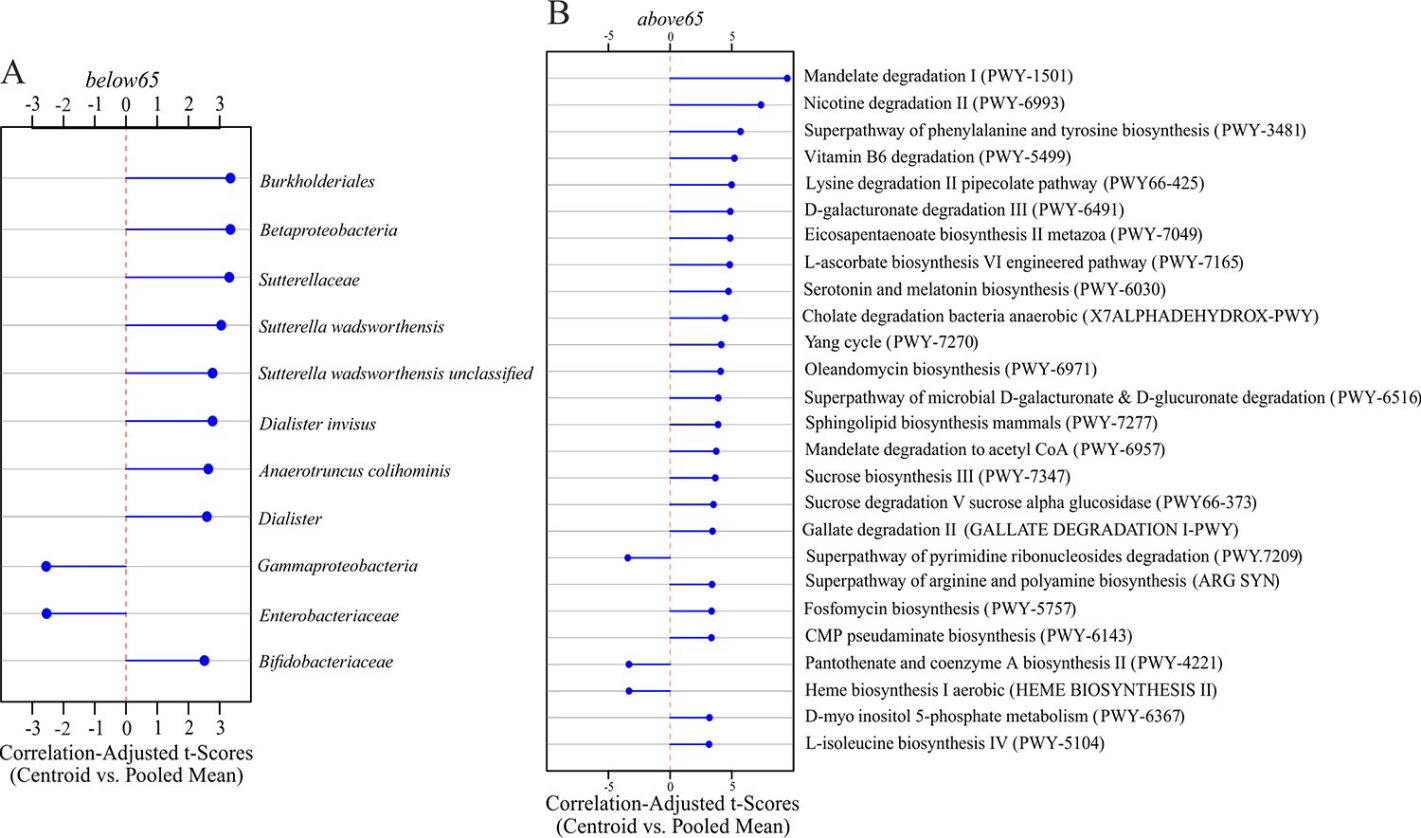
Top 11 bacteria and top 26 pathways according to CAT-scores. The (A) top ranked 11 bacteria and (B) top ranked 26 pathways according to CAT-scores. The length and direction of the blue bars indicate the influence of a given biomarker on the discriminative power of the model. A: The order *Burkholderiales* within the class *Betaproteobacteria* have the highest potential for separation of the age groups with a positive CAT-score indicating an over-representation in the *below65* group. While both, the family *Enterobacteriaceae* within the class *Gammaproteobacteria* have a negative CAT-score indicating an over-representation in the *above65* group. B: Most of the pathways shown have a higher relative abundance in the elderly population with mandelate degradation (PWY-1501) contributing most to group separation. *above65*: patients aged 65 years and above (n = 145), *below65*: patients below 65 years of age (n = 133). CAT-scores, correlation adjusted T-scores.

**Fig 6:**
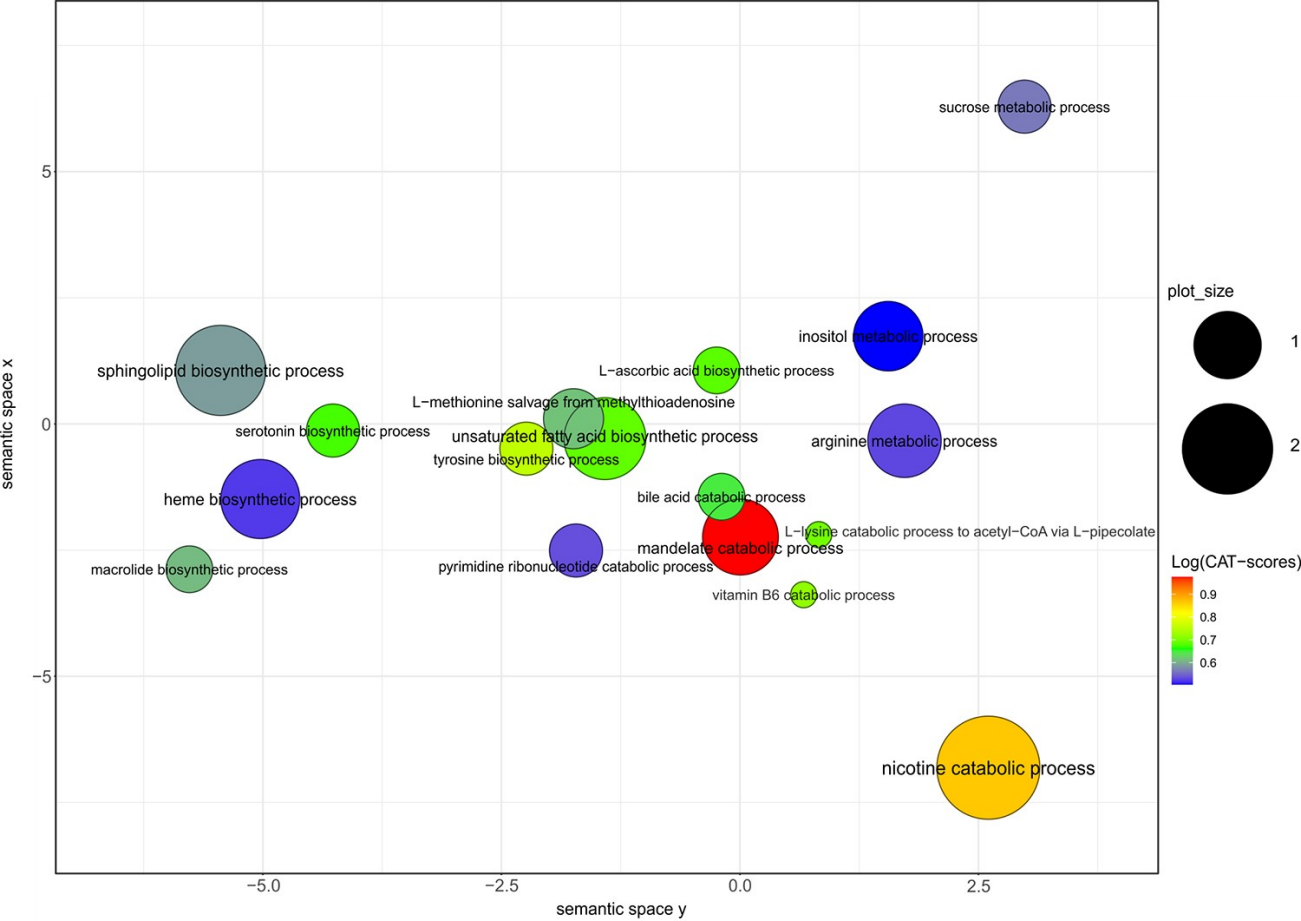
Scatterplot of the top 26 pathways based on GO terms. Cluster representatives (i.e. GO terms remaining after redundancy reduction by REVIGO) are shown. Distances between bubbles represent the semantic similarities between the GO terms, bubble position is determined by application of multidimensional scaling to a matrix of the GO terms’ semantic similarities (the lower the distance, the more similar the terms). The axes values have no intrinsic meaning. Bubble size indicates the frequency of the GO term in the underlying GO database. Logarithmized CAT-scores are visualized using a color gradient from red to blue. GO, gene ontology; CAT-scores, correlation adjusted T-scores.

To further illustrate the age-dependency of the intestinal microbiome, machine learning approaches were applied to identify potential biomarkers for the age groups. Model selection of several common machine learning algorithms for classification revealed Random Forest as most suitable for the data set since it showed significantly better performance for both, microbial and pathway abundances, compared to the other algorithms tested (S1 Fig). Furthermore, to increase the accuracy of Random Forest, hyperparameters were tuned, suggesting setting the number of variables used in each split (mtry) to 11 and the number of generated trees in the forest (nTree) to 2000 for taxonomic features and setting mtry to 26 and nTree to 2000 for functional features, respectively. After tuning, the model was trained and evaluated by 10 fold cross-validation (Table 2). While accuracies between 0.72 and 0.86 mean that between 72 and 86% of the features are classified correctly by the Random Forest approach, kappa between 0.44 and 0.71 indicates moderate to substantial agreement according to Landis and Koch [36]. To further assess the classifier’s performance, ROC curves were computed (Fig 4). With an AUC of 0.91 for microbial abundances (Fig 4A) and 0.77 for pathway abundances (Fig 4B), the discrimination capacities of the models to distinguish between age groups is good. Finally, to identify those taxonomic and functional features contributing most to group separation, shrinkage discriminant analysis based on CAT-scores was applied (Fig 5). According to mtry in Random Forest, 11 bacterial groups (Fig 5A) and 26 pathways (Fig 5B) with the highest CAT-scores were considered. GO term based clustering revealed that age-dependent functional features of the microbiome are mainly involved in biosynthetic processes of heme, sphingolipid,unsaturated fatty acids and nicotine catabolic process (Fig 6).

**Table 2:** Performance of random forest on microbial and pathway abundances.

	Microbial abundances	Pathway abundances
Sample size	278	277
Accuracy	0.755	0.724
Kappa	0.507	0.442

The model was evaluated by ten times repeated 10-fold cross-validation. Accuracy shows the number of correctly classified features out of all features, while kappa depicts the accuracy normalized at the baseline of random chance on the dataset.

## Discussion

In this study, the effects of age on the intestinal microbiome and its functional profile were investigated. The intestinal microbiome is relatively stable during adulthood [4], but several studies reported aberrations in older individuals [11, 37]. In accordance to previous studies [35, 38], the microbiomes in this cohort were dominated by the phyla *Bacteriodetes* and *Firmicutes* (Fig 1). However, differences in relative abundances of microbes between the age groups *below65* and *above65* compared in this study, have been identified by two independent approaches, by MaAsLin (Fig 3C) and by machine learning algorithms using Random Forest (Fig 5A). Using PCA, the group separation may in part be due to outliers since most of the data clusters together (Fig 3A and 3B). However, both approaches showed that the class *Betaproteobacteria* as well as its order *Burkholderiales* and the family *Sutterellaceae* including the species *Suturella wadsworthensis* had a higher relative abundance in the *below65* age group, whereas the class *Gammaproteobacteria* and its family *Enterobacteriaceae* had a higher relative abundance in the *above65* age group compared to the respective other group.

Although being in relatively low abundance compared to *Bacteriodetes* and *Firmicutes*, alterations in the phylum *Proteobacteria* may have a considerable effect on human health since an elevated prevalence of *Proteobacteria* has been proposed as a diagnostic marker for an unstable intestinal microbial community called dysbiosis and for risk of disease [38–40]. Moreover, the family *Bifidobacteriaceae* and its genus *Bifidobacterium* including the species *Bifidobacterium adolescentis* were of higher relative abundance in the *below65* compared to the *above 65* age group. Since it has been proposed in many studies that *Bifidobacterium* can be used as probiotic to alleviate various disease by modulating the intestinal microbial composition [41], reduced *Bifidobacteriaceae* may be used as marker for dysbiosis and disease progression in the elderly. Furthermore, increasing proportions of *Enterobacteriaceae* as observed in the *above65* group, including *Klebsiella spp*., *Enterobacter aerogenes* and *Escherichia coli*, were also observed in patients suffering from atherosclerotic cardiovascular disease [42]. Bacteria of the genus *Klebsiella* have been observed in higher abundances in patients with hypertension and pre-hypertension [43]. Thus, the family *Enterobacteriaceae* and especially its genus *Klebsiella* may be a marker for disease in the elderly.

Concerning taxonomic features, the two approaches applied for age group separation in this study, resulted in similar results with some exceptions at low taxonomic levels. On the level of functional features, machine learning algorithms assisted in reducing the data set into pathways with the highest discriminative potential in the prediction model of the cohort. Whereas MaAsLin resulted in 305 significantly different pathways between *below65* and *above65* (S2 Table), the use of Random Forest approach allowed to reduce this list to the 26 pathways contributing most to group separation (Fig 5B). This shows that machine learning is a powerful tool to find key differences in the data set. However, there are also disadvantages: Unlike to simple p-value test, relevance scores used to highlight multivariate interacting effects in machine learning approaches are usually difficult to interpret [44]. Moreover, through bootstrapping of the dataset some samples may be lost due to random sampling, leading to possible neglect of crucial data such as outliers. Using machine learning to develop classifiers for disease detection has the advantage in its non-invasive nature, but it is crucial to use attributes resulting in classifiers with reasonable predictive value for disease instead of confounding variables [45]. Therefore, combining different approaches, termed hybrid machine learning, may result in more stable and better predicting algorithms to detect potential biomarkers [46]. In this study, only one machine learning approach (Random Forest that showed the best performance in the prediction model, S1 Fig) was applied, but a second independent algorithm based on linear models (MaAsLin) was used for data analysis. This may mutually exclude the drawbacks of the two approaches and generate more robust results.

In this study, the highest discriminative power in the proposed model was attributed to 26 pathways that are mainly involved in biosynthetic processes of heme, sphingolipid, unsaturated fatty acids and nicotine catabolic process (Fig 6). Many studies have shown associations between the Firmicutes to Bacteroidetes ratio and several diseases such as obesity [47] and also including age-dependent diseases such as age-related macular degeneration [48]. Since the order Selenomonadales is part of the phylum Firmicutes, the positive correlation found between Selemondales and the BMI in this cohort may point to these associations. Moreover, the fatty acid profile is linked to various metabolic disorder including obesity [49]. Thus, there may be an age-associated connection between the Firmicutes to Bacteroidetes ratio, fatty acid synthesis and several diseases such as obesity. Molano et al. showed age-dependent changes in the sphingolipid composition of immune cells, resulting in immune dysregulation [50]. Thus, the age-dependent sphingolipid biosynthesis by gut microbes found in this study may be linked to diminished functions of the immune system and associated diseases in the elderly. Moreover, in agreement with our data, it has been shown in a murine model of Western diet in the USA that the microbiome affects both, the plasma fatty acids and the liver sphingolipids [51]. Since the heme metabolism may be altered in age-related diseases, probably involving oxidative damage that is triggered by free heme, and since the biosynthesis of heme requires Vitamin B6 [52], the age-dependent biosynthesis of both, heme and Vitamin B6, found in this study may be a trigger for age-related diseases. Previous studies have shown that nicotine exposure alters the intestinal microbiome secondary to diet [53] and in our cohort, we identified an age-associated nicotine catabolic process, supposing an altered degradation of nicotine in the elderly in association with the taxonomic composition of the microbiome.

## Conclusions

This study revealed taxonomic and functional features of the intestinal microbiome associated with age and with a potential link to age-associated diseases in humans. Therefore, these results may have important implications on preventive strategies for degenerative processes occurred in the elderly by using microbiome-altering interventions. Given the significant differences found between the age groups in this study by two independent approaches, advising to use age-matched groups for unbiased metagenomic data analysis in further studies and to consider the drawbacks of the algorithms used for data analysis, thus, probably applying a second independent approach to generate robust results.

## Supporting information

S1 Table Taxonomic characterization of the intestinal microbiome by MetaPhlAn2. The most abundant phyla are highlighted in red, the most abundant classes in green, the dominating genera in purple and the most abundant species in yellow.
(XLSX)

S2 Table Distinct functional composition between age groups. Correlation between functional features and age (MaAsLin, q < 0.2). Positive correlations (orange) imply higher abundance in *below65*, whereas negative correlations (blue) imply higher abundance in *above65*.
(XLSX)

S1 Fig Model selection for machine learning algorithm based on microbial abundances. RF, Random Forest; SVM, support vector machine; CART, Classification and Regression Trees; KNN, K-Nearest Neighbor.
(TIF)
