# Supplementary figures and images for: The importance of age in compositional and functional profiling of the human intestinal microbiome
Source: PLoS One. 2021 Oct 18;16(10):e0258505. doi: 10.1371/journal.pone.0258505 (PMC8523055; doi:10.1371/journal.pone.0258505)

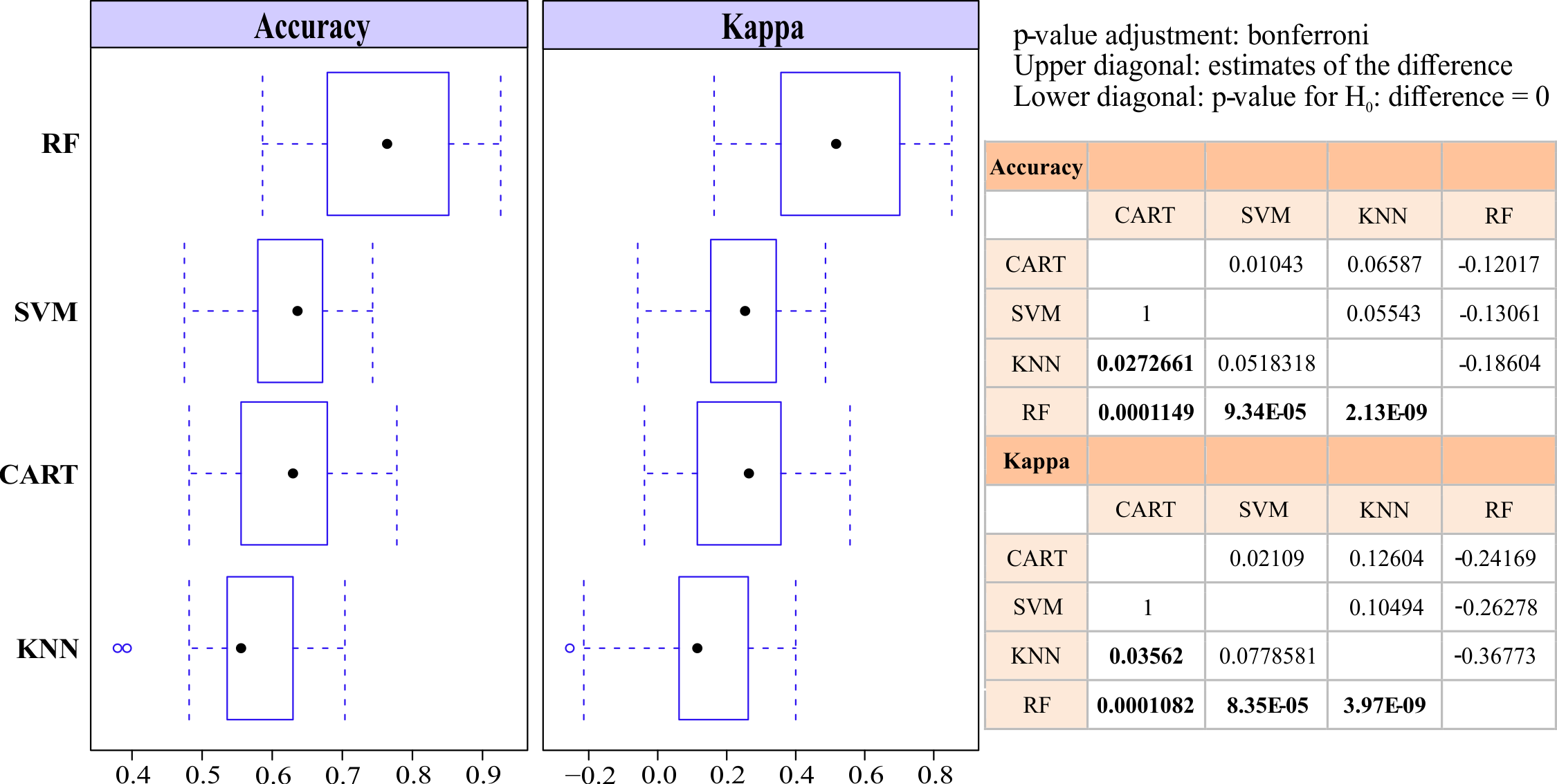

Supplement: S1 Fig — RF, Random Forest; SVM, support vector machine; CART, Classification and Regression Trees; KNN, K-Nearest Neighbor. (TIF) [file pone.0258505.s003.tif]
